# Automated BigSMILES conversion workflow and dataset for homopolymeric macromolecules

**DOI:** 10.1038/s41597-024-03212-4

**Published:** 2024-04-11

**Authors:** Sunho Choi, Joonbum Lee, Jangwon Seo, Sung Won Han, Sang Hyun Lee, Ji-Hun Seo, Junhee Seok

**Affiliations:** 1 School of Electrical Engineering, Korea University, Seoul, South Korea; 2 Department of Materials Science and Engineering, Korea University, Seoul, South Korea; 3 School of Industrial Management Engineering, Korea University, Seoul, South Korea

**Keywords:** Polymer chemistry, Polymer characterization, Polymerization mechanisms

## Abstract

The simplified molecular-input line-entry system (SMILES) has been utilized in a variety of artificial intelligence analyses owing to its capability of representing chemical structures using line notation. However, its ease of representation is limited, which has led to the proposal of BigSMILES as an alternative method suitable for the representation of macromolecules. Nevertheless, research on BigSMILES remains limited due to its preprocessing requirements. Thus, this study proposes a conversion workflow of BigSMILES, focusing on its automated generation from SMILES representations of homopolymers. BigSMILES representations for 4,927,181 records are provided, thereby enabling its immediate use for various research and development applications. Our study presents detailed descriptions on a validation process to ensure the accuracy, interchangeability, and robustness of the conversion. Additionally, a systematic overview of utilized codes and functions that emphasizes their relevance in the context of BigSMILES generation are produced. This advancement is anticipated to significantly aid researchers and facilitate further studies in BigSMILES representation, including potential applications in deep learning and further extension to complex structures such as copolymers.

## Background & Summary

The simplified molecular-input line-entry system (SMILES) is a chemical representation developed in 1988 to express chemical formulas in ASCII strings^1^. The underlying rules in SMILES are relatively intuitive; adjacent atom strings are also closely connected in actual structures, and a fork, which is divided into two or more branches, can be indicated through parentheses, and a ring notation such as a benzene ring, can be expressed through numbers. Although the International Chemical Identifier (InChI)^2,3^, which allows chemical line notation in a mechanism not similar to SMILES, is also widely used in the overall field of chemistry, its complex and incomprehensible syntax has led to InChI being considered to be not as efficient as SMILES, and it has limited applications in the field of artificial intelligence^4–6^. Due to its ability to express chemical structures as strings using intuitive syntax and its ease of handling via various tools, SMILES has been extensively applied to polymer property analyses and predictions by machine learning and deep learning algorithms^5–18^.

However, SMILES representation suffers from certain limitations in predictive and generative studies^7,19–26^. Because SMILES is specialized in representing small molecules, additional notation system is required for the representation of macromolecules such as polymers. The representative chemical line notations applying similar creation mechanisms that have been developed since SMILES include SMARTS^27^, SELFIES^28^, DeepSMILES^29^, and BigSMILES^30^. Especially noteworthy is that BigSMILES, specialized in representing macromolecules, is anticipated to overcome the limitations in artificial intelligence research by encompassing three key pieces of information in addition to what SMILES provides^18,19,25,31^. These include the principle of polymerization through reactions, the indication of the significance of both head-to-tail and tail-to-tail configurations, and the information to distinguish between two reactive end groups in step-growth polymerization. BigSMILES enables the provision of such information by incorporating several rules utilizing special characters in addition to the SMILES rules.

Numerous researchers have expressed a keen interest in employing BigSMILES representations in polymer research. However, there remains a paucity of machine learning or deep learning investigations in this domain^32^. This limitation stems largely from the absence of readily available source codes or software programs to generate BigSMILES data without environmental restrictions compared to other representation methods. Therefore, in the realm of artificial intelligence research, it remains imperative that all data must be meticulously curated manually by domain experts.

**Fig. 1:**
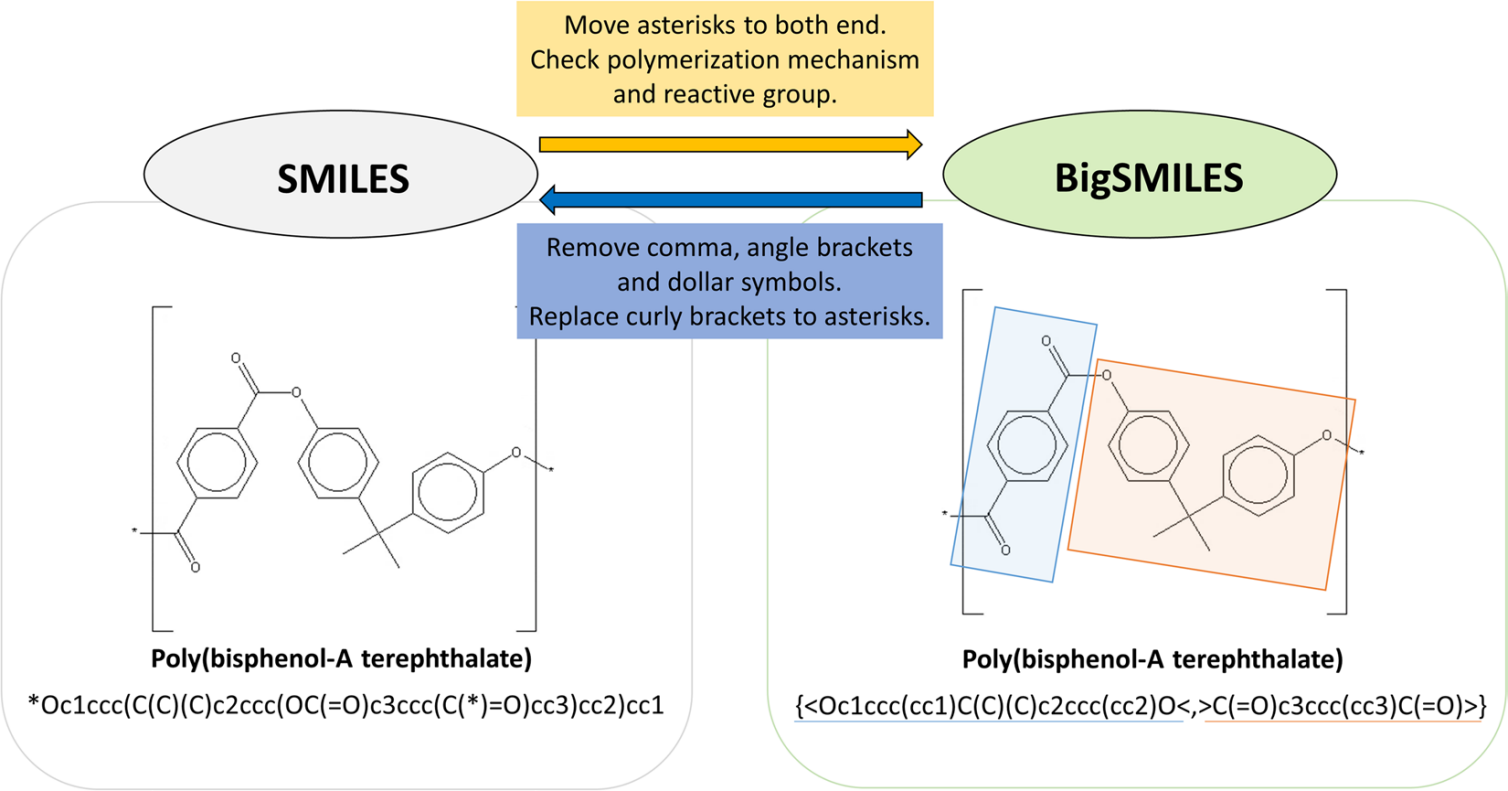
Example of mutual conversion between SMILES and BigSMILES.

Thus, this study propose an automated system designed specifically for generating BigSMILES from SMILES representations of homopolymers, with a primary emphasis on simpler structures that are readily amenable to the identification of conversion rules. The proposed system has been used to create dataset files applying BigSMILES representations to 4,927,181 records, and is ready for immediate use in various research fields. We aimed to enable interchangeable creation of BigSMILES representations from SMILES data without manual intervention, as shown in Fig. 1. This advancement can streamline the research efforts focused on the BigSMILES representation of homopolymers, potentially paving the way for deep learning and other related studies in this domain. We hope that this development will stimulate active research endeavors in the future, not only for homopolymers but also for more intricate and complex structures such as copolymers.

## Methods

### BigSMILES polymer representation in homopolymers

The BigSMILES representation adheres to the fundamental principles of SMILES and is applicable to the depiction of all polymers, not exclusively homopolymers. BigSMILES inherits both non-unique and unambiguous features of SMILES. Uppercase and lowercase letters represent non-aromatic and aromatic atoms, respectively. Special characters are employed to signify the bonding relationships between atoms. Parentheses indicate side chains in the presence of branching points in the bond relationships. Ring structures such as benzene are denoted using numeric values. For chemical substances with multiple rings, distinct numbers are used to distinguish ring structures. An asterisk (*) is often used as a wildcard symbol to represent any element or group of elements, or used as a symbol of polymerization point in the constitutional repeating unit. However, in this study, we considered the asterisks only as polymerization points because the information obtainable solely from SMILES input is limited. Additionally, as applying automated general rules for repeating units with multi-atom connections is challenging^30^, all of the SMILES representations of repeating units of homopolymers, which is a primary focus of this study, all SMILES representations feature two polymerization points. Because homopolymers are formed by the repetitive linkage of identical repeating units, the same polymer segment is assumed to be attached to each point.

Expanded from the foundational SMILES syntax, the BigSMILES syntax introduces the use of curly brackets to represent polymeric fragments. For homopolymers, the polymerization point notations are substituted with curly brackets. However, it is challenging to adapt this to the existing SMILES representation; an asterisk, which signifies the polymerization point, must be positioned at both ends of the SMILES expression. Because most canonical SMILES forms do not place asterisks at either end, this study developed an algorithm to reposition asterisks at both ends without altering the original structure of the polymeric fragment to be depicted.

**Fig. 2:**
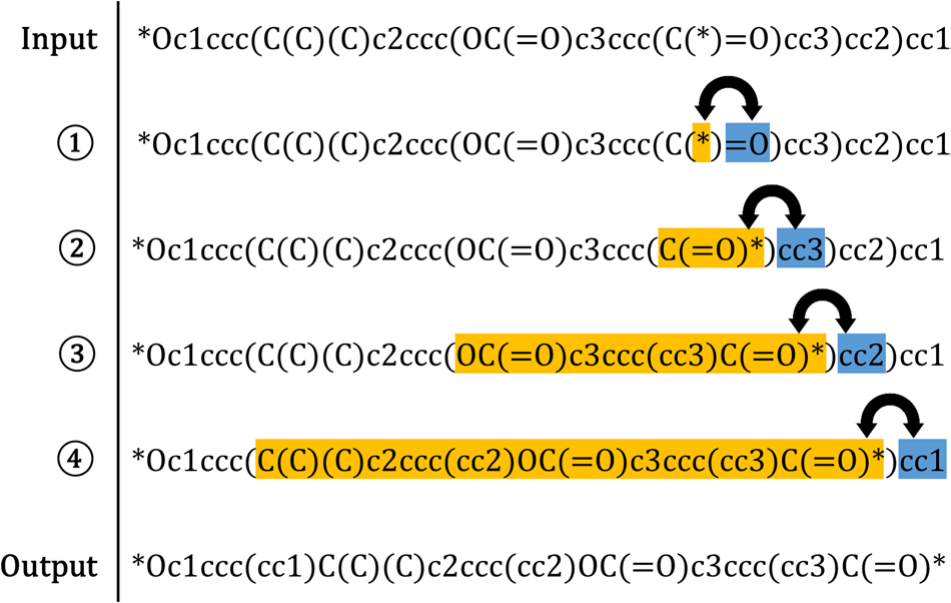
Example of the permutative process of moving the asterisks to either end when entering the canonical SMILES of Poly(bisphenol-A terephthalate). The portion identified as the main chain varies with each permutation; however, these SMILES notations represent the same polymer while preserving its structural integrity, Poly(bisphenol-A terephthalate).

The asterisk, which was previously treated as a branch and located in the middle of the original canonical SMILES, is swapped with the remaining portion located to the right of the parentheses. This repositioning ensures that the remaining portion is recognized as a branch. This approach exploits the fact that, in the SMILES expression, the branched parts are enclosed within parentheses, while the main structure is located outside them. Throughout this process, a careful attention must be paid to ring numbering and isotopes to ensure that the altered representation is logically consistent with the original repeating units. Consequently, the sequence of elements is rearranged, with the main chain of the repeating unit placed outside all parentheses, and the side chains defined within them. Figure 2 illustrates a permutative process applied to Poly(bisphenol-A terephthalate). The code implementing this methodology is provided in the Python programming language to facilitate reproducibility.

Furthermore, a paramount distinction between BigSMILES and SMILES is the direct incorporation of information regarding the polymerization mechanism into the representation. This is achieved by introducing special symbols that are not used in SMILES— angle brackets (<, >), dollar signs ($), and a comma (,). For homopolymers, these angle brackets and dollar signs are positioned adjacent to curly brackets.

The dollar symbol signifies monomers engaged in polymerization through AA type bonding, encompassing both chain-growth and addition polymerization. Conversely, angle brackets indicate the monomers involved in polymerization through AB type bonding, encompassing step-growth and condensation polymerization. Thus, the presence of the dollar sign within the BigSMILES expression provides an immediate insight that the corresponding polymer undergoes AA type polymerization, obviating the need for further analysis. Consequently, this feature simplifies the task of discerning variances in polymer characteristics due to different polymerization processes compared to alternative representation methods.

**Fig. 3:**
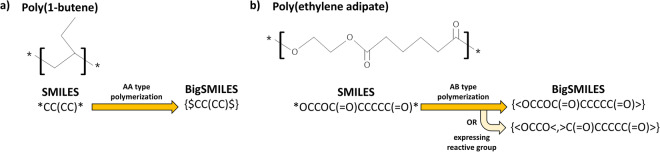
Examples of SMILES to BigSMILES conversion results. (**a**) AA type polymerization compound Poly(1-butene). (**b**) AB type polymerization compound Poly(ethylene adipate). For AB type polymers, two expression techniques, i.e., displaying only the AB-type and displaying the reactive group, are possible.

The adoption of BigSMILES enables swift differentiation between AA and AB type polymers. However, for AB type polymers that form a condensation polymerization with different monomers, providing additional information regarding the two reactive end groups can be advantageous, as it offers insights into the two-unit monomers responsible for step-growth polymerization. For homopolymers, if two reactive groups are identified in the SMILES representation, BigSMILES incorporates a “<, >” symbol between these groups. For instance, consider Poly(ethylene adipate), represented as “OCCOC( = O)CCCCC( = O)” in SMILES. While BigSMILES representation may initially portray Poly(ethylene adipate) as “{ < OCCOC( = O)CCCCC( = O) >}” with an AB polymerization type, it can also be encoded as “{<OCCO <, > C( = O)CCCCC( = O) >}” to distinctly denote the presence of reactive end groups. Figure 3 presents a practical illustration of the conversion from SMILES, showing the repeating units of the two polymers to BigSMILES, demonstrating the capability of expressing reactive groups in AB type polymers.

Nonetheless, even when the provided SMILES repeating unit data are categorized as either AA or AB type, actual polymerization types may undergo alterations under specific synthesis conditions, such as the application of a catalyst. In these exceptional scenarios, supplementary adjustments become imperative subsequent to employing the automated BigSMILES conversion code outlined in this study. This is essential in that the automation code discerns the polymerization type based solely on the provided repeating unit SMILES, without considering the impact of specialized synthesis conditions.

Following the publication on the development of BigSMILES syntax, subsequent work has extended the BigSMILES language to include methods and syntax for canonicalization and noncovalent bonding^33–35^. As a result, the latest BigSMILES line notation differs slightly from the rules established in the initial BigSMILES publication and incorporates additional features. However, in this study, we adhere to the syntax established in the original BigSMILES publication, acknowledging the possibility that rules may continue to be added or modified in the future.

### Workflows

**Fig. 4:**
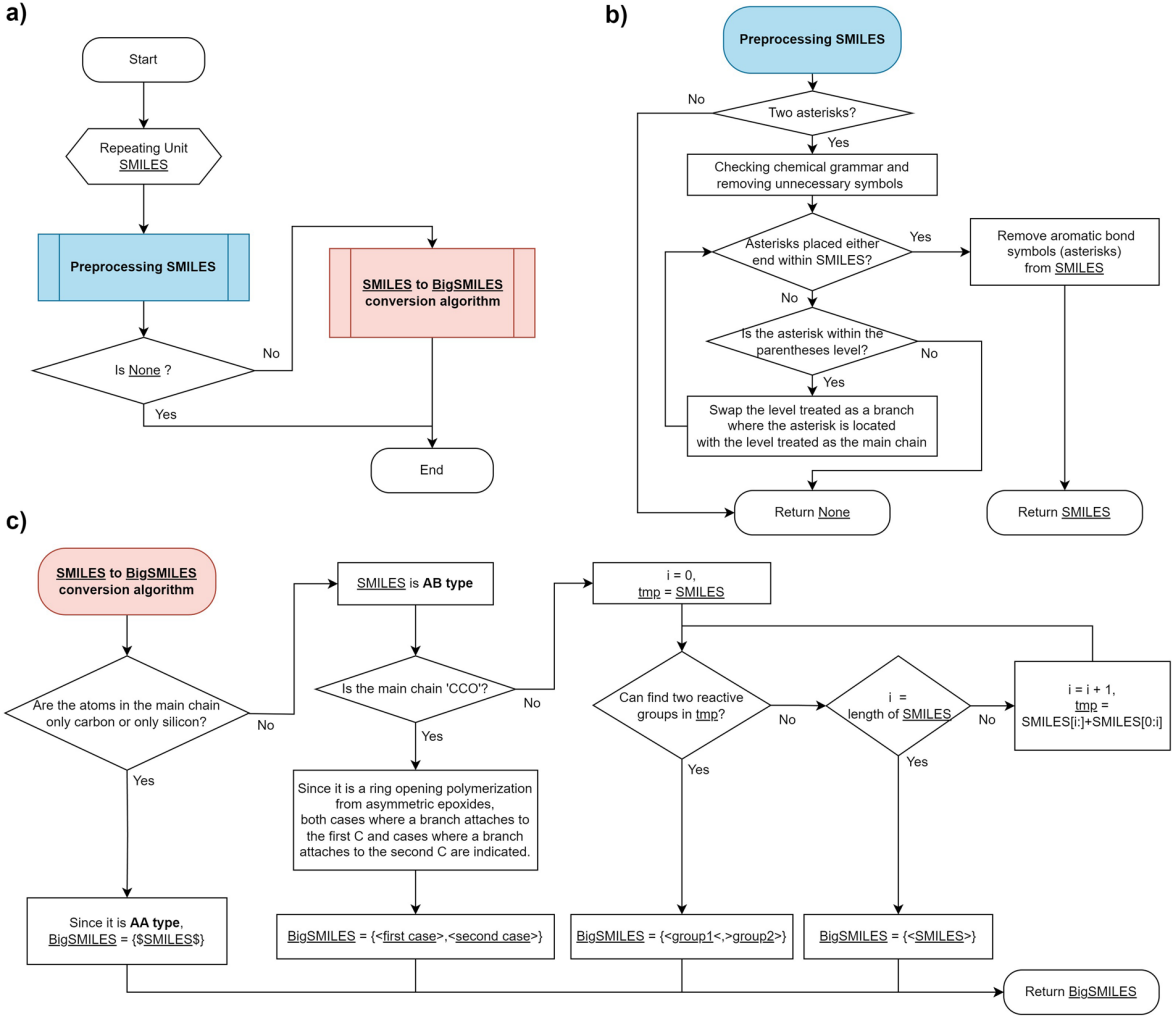
Workflows of SMILES to BigSMILES conversion algorithm. (**a**) Overall workflow. (**b**) Preprocessing process workflow. (**c**) SMILES to BigSMILES conversion process workflow.

The workflows illustrated in Fig. 4 encapsulates the comprehensive processes involved in the conversion of SMILES into BigSMILES. The conversion between SMILES and BigSMILES yields distinct representations of the same substance. In essence, it entails converting a substance represented in SMILES into BigSMILES notation by deriving three pieces of information: the principle of polymerization through reactions, an indication of the significance of both head-to-tail and tail-to-tail configurations, and information to distinguish between two reactive end groups in step-growth polymerization.

The overall workflow entails an initial preprocessing stage, wherein polymerization point symbols, represented by asterisks (*), are positioned at both ends of the SMILES for each repeating unit SMILES input. Subsequently, the preprocessed SMILES are transformed into BigSMILES. If the asterisk is not relocated to either end of the SMILES during the preprocessing stage, the conversion to BigSMILES cannot be successfully achieved.

The procedure of relocating the asterisk to both ends is reiterated until all asterisks are successfully positioned at both ends of the SMILES, as illustrated in the example provided in Fig. 2. Cases in which the number of polymerization points is not equal to two are filtered out at this stage. To check the chemical grammar, we seamlessly implement it in Python, the language used for this workflow, utilizing the chemical representation package RDKit^36^. The process involves using RDKit to ensure that the SMILES representations adhere to chemical grammar, confirming their validity. To remove unnecessary symbols, adjustments were made to eliminate redundant SMILES representations and streamline the BigSMILES conversion process. Examples of this include removing the “–” symbol for a single bond, which is usually omitted. If all the asterisks are located outside the parentheses but not positioned at either end within the SMILES, attempts to relocate them to both ends will be futile. This is because there exists no primary chain to modify their placement. Once the asterisks are repositioned at both ends, these symbols are removed, and the subsequent step proceeds assuming that both ends of the preprocessed SMILES represent polymerization points.

Once the preprocessed SMILES is generated under the assumption of a polymerization point at both ends, it is fed into the conversion algorithm. Drawn from the insights and data presented in the research on BigSMILES, the polymer is classified as follows: If the main chain of the SMILES exclusively comprises carbon or silicon atoms, it is categorized as an AA type because such polyolefins and polycarbosilanes are commonly prepared by Ziegler–Natta polymerization or ring-opening polymerization, respectively. Conversely, unless this condition is satisfied, it is designated as the AB type. As explained earlier, this standard is not inflexible and may be subjected to alterations based on factors, such as the utilization of specialized reactions.

**Fig. 5:**
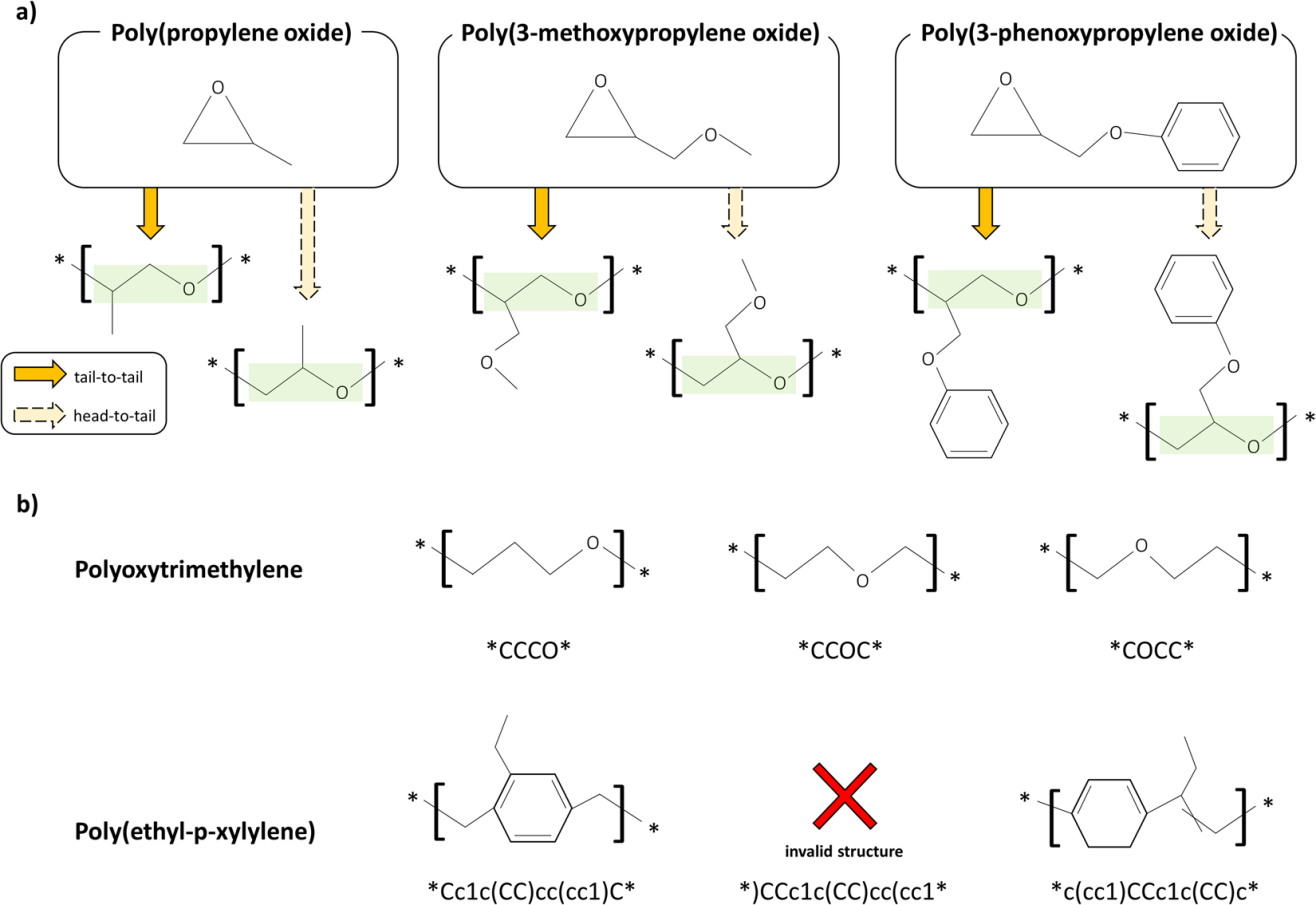
Examples to illustrate intermediate steps in the workflows. (**a**) Examples of ring opening polymerization from asymmetric epoxides. The main chain of the repeat unit is made up of CCO. (**b**) Examples of parallel displacement in SMILES notation.

If the classification into the AA type is confirmed, processing can be concluded. However, in cases classified as AB type, it is essential to verify whether the specific criteria are met. The primary criterion involves identifying situations wherein both head-to-tail and tail-to-tail configurations are significant. In the BigSMILES study, these attributes are specifically exemplified within the Propylene oxide series, characterized by their formation through the ring opening polymerization from asymmetric epoxides. To consider these attributes in SMILES representations wherein the structure prior to the reaction is unknown, we identify SMILES sequences characterized by a main chain composed solely of “*CCO*” and consider them as exceptions. This entails a BigSMILES representation that expresses both head-to-tail and tail-to-tail configurations. An illustrative example is shown by the three propylene oxide series examples in Fig. 5.

The next aspect under consideration is the partitioning of AB type repeating unit into two reactive groups. This procedure is initiated with preprocessed SMILES and involves stepwise repositioning of polymerization points. In simpler terms, this involves sequentially shifting each SMILES notation until parallel displacement is achieved throughout the length of that SMILES notation. This approach draws an inspiration from the fact that, as presented in Fig. 5, the SMILES notation of Polyoxytrimethylene represents the same polymer even when the structural elements near the polymerization point, such as “*CCCO*”, “*CCOC*”, and “*COCC*”, are moved in parallel. However, the unit of displacement is a single character, and occasional SMILES syntax violations may occur when conducting parallel displacements, such as the sole movement of parentheses or numbers. When parentheses are shifted, there is a potential risk of encountering invalid syntax issues. Similarly, when numbers are shifted, there is a probability of representing a structure different from the original structure. In such instances, it is impossible to identify two reactive groups from the outset; consequently, these cases are excluded from consideration. Typically, these exclusions occur more than the total number of parentheses plus the number of digits for a single SMILES. Once a single unit undergoes parallel displacement without anomalies, the subsequent step involves inspecting whether, within this temporary SMILES, two specific structures from the predefined forms of reactive end groups seamlessly fit together. The predefined forms of reactive end groups used in this step are primarily adopted from existing literature, with some additions incorporated during the ground-truth creation and manual verification stages of this study. Throughout this process, upon discovering the presence of two reactive groups, the transformation proceeds to represent both groups within the AB type notation, prompting the cessation of the parallel displacement process. However, if the search reached the last iteration without discovering these groups, it simply confirms the presence of the AB type. In some chemical instances, the absence of two reactive groups can occur, either as a result of the non-existence of the homopolymer or the complexity of its structure, rendering it difficult to identify such groups.

### Data acquisition

A homopolymer dataset comprising 4,927,181 records was obtained from three distinct studies. All of the data sources explicitly stated that the data were available for academic use. Among these, 966 records included both SMILES representation data and their corresponding glass transition temperature values. These records were provided in the form of supplementary information in their respective papers. Within this subset, 304 records represented the experimentally verified temperature values derived from the entries of the standard dataset of the glass transition temperature of linear polymers^30,37^; the remaining 662 records were derived through machine calculations^7^. As will be discussed in the Technical Validation section, the initial 304 data records were manually validated once and were found to differ from the original source by about eight records. The remaining 4,926,212 records consisted solely of SMILES representations generated by systematically combining the chemical fragments extracted from synthesis-proof polymers^8^. These records were provided in the form of a Zenodo database^38^. Even though the data provided in the database total approximately 100 million records, because the feasibility of synthesizing these data into actual polymers has not been empirically demonstrated, we decided to convert only records with SMILES lengths of 85 characters or less to reflect the fact that excessively long repeating unit structures might be less practically feasible.

## Data Records

The complete dataset of 4,927,181 homopolymers with BigSMILES representations can be accessed from Figshare^39^. All dataset files are available in comma-separated values (csv) format. Notably, all of the BigSMILES values present in the dataset were automatically generated using the conversion algorithm. The detailed description of the dataset with and without the glass transition temperature is presented below. Each dataset exists in the “with_Tg” and “without_Tg” folders in Figshare.

### Dataset with glass transition temperature (966 records)

- **JCIM_sup_bigsmiles.csv** is a csv file containing 662 records of machine-derived homopolymer structures and glass transition temperatures. All 662 generated BigSMILES records were verified by a chemist. The file is structured with the following fields:
  1. *SMILES* field indicates the homopolymer’s repeating unit SMILES representation for each row;
  2. *BigSMILES* field indicates the homopolymer’s BigSMILES representation for each row;
  3. *Tg (C)* field represents the °C temperatures of the data collected for each row.
- **Bicerano_bigsmiles.csv** is a csv file containing 304 records of experimentally verified homopolymer structures and glass transition temperatures. All 304 generated BigSMILES records were verified by a chemist. Since the data has been constructed from existing polymer structures, the information on the polymer name was also included. The remaining fields are the same as those shown in the file above. The file is structured with the following fields:
  1. *Polymer name* field represents the existing polymer structure name for each row;
  2. *SMILES* field;
  3. *BigSMILES* field;
  4. *Tg (K) exp* field represents the temperature in Kelvin of the data collected for each row.

### Dataset without glass transition temperature (4,926,212 records)

- **polyBERT_len85_######.csv** in “without_Tg” folder comprising 4,926,212 records, is segmented into a total of 50 csv files. Each file accommodates data in batches of 100,000 records and is stored as an individual file with sequential nomenclature (polyBERT_len85_0.csv, polyBERT_len85_100000.csv, polyBERT_len85_200000.csv, etc.) The final file, polyBERT_len85_490000.csv, has 26,212 records. The dataset contains only the BigSMILES field. The corresponding SMILES data can be accessed on the Zenodo database^38^.

## Technical Validation

To verify the reliability of the dataset and workflow, three validation procedures were conducted. The first objective was to verify the accuracy of the conversion algorithm. The second focused on ensuring that there was no loss of information or disruption in the conversion of the representation methods resulting from the algorithm. Finally, we verified the robustness of the algorithm, ensuring that it performed consistently regardless of the input. The first and second steps of this process involved testing the success rate of the mutual conversion between SMILES and BigSMILES, as shown in Fig. 1. These three technical validations were executed through Python programming code. For clarity, all steps in the process were executed algorithmically, and any aspects that require manual confirmation were initially verified by a data scientist who was aware of the underlying encoding principles of SMILES and BigSMILES and had conducted workflows. The next step was subsequent validation based on the chemical structure by a chemist. Thus, for each technical step that required manual validation, two-stage verification was conducted from data and chemical perspectives, respectively.

The first validation procedure involved verifying whether the BigSMILES ground-truth provided by the original data source matched the BigSMILES representation generated using our conversion algorithm. Because the dataset with BigSMILES representation was primarily sourced from a paper introducing BigSMILES, we conducted a validation process on 304 records to ensure the precision of the conversion.

Our initial step involved verifying the integrity of 304 BigSMILES representations to ascertain their suitability as the ground-truth. We cross-referenced the BigSMILES representations with the polymer structure images provided in the respective previous study to detect typographical errors. We identified discrepancies in the BigSMILES representations of eight polymer structures, as outlined in Table 1. The index columns corresponded to those in the provided dataset. Among these, three records were found to contain structural errors that were not logically feasible, whereas five records were corrected because they originally represented different polymers. These eight errors were rectified and adopted as the ground-truth. The names following the colon after “Perfluoropolymer” in the table represent the IUPAC names^40^ of the respective polymers. They have been added as clarifications to avoid confusion with polymers named using common nomenclature. The IUPAC names corresponding to each molecule were generated using a IUPAC name generation toolkit named Marvin Suite^41^ and then verified by a chemist. Thus, these two cases of “Perfluoropolymer” were checked for anomalies and added to the table.

**Table 1:** List of corrected errors in BigSMILES present during data collection.

Index	Polymer name	Reference SMILES	Obtained BigSMILES	Corrected BigSMILES
28	Poly(3-methoxypropylene oxide)	*CC(COC)O*	{<CC(OC)O>, <C(OC)CO>}	{<CC(COC)O>, <C(COC)CO>}
65	Perfluoropolymer: poly[2‐(1‐{[1,1,2,2,3,3,4,4,5,5,6,6‐dodecafluoro‐6‐(2,2,2‐trifluoroethoxy)hexyl]oxy}‐1,2,2,2‐tetrafluoroethyl)‐4‐methyl‐1,3,5‐triazine]	*c1nc(C)nc(C(F)(OC(F)(F)C(F)(F)C(F)(F)C(F)(F)C(F)(F)C(F)(F)OC(*)C(F)(F)F)C(F)(F)F)n1	{<C(C(F)(F)F)OC(F)(F)C(F)(F)C(F)(F)C(F)(F)C(F)(F)OC(F)(C(F)(F)F)c1nc(nc(C)n1)>}	{<c1nc(C)nc(n1)C(F)(C(F)(F)F)OC(F)(F)C(F)(F)C(F)(F)C(F)(F)C(F)(F)C(F)(F)OC(C(F)(F)F)>}
69	Perfluoropolymer: poly[2‐(2,2,2‐trifluoro‐1‐{[1,1,2,2,3,3,4,4,5,5,6,6,7,7‐tetradecafluoro‐7‐(2,2,2‐trifluoroethoxy)heptyl]oxy}ethyl)‐4‐(trifluoromethyl)‐1,3,5‐triazine]	*C(C(F)(F)F)OC(F)(F)C(F)(F)C(F)(F)C(F)(F)C(F)(F)C(F)(F)C(F)(F)OC(C(F)(F)F)c1nc(nc(C(F)(F)F)n1)*	{<C(C(F)(F)F)OC(F)(F)C(F)(F)C(F)(F)C(F)(F)C(F)(F)C(F)(F)C(F)(F)OC(C(F)(F)F)c1nc(nc(C(F)(F)F)n)>}	{<C(C(F)(F)F)OC(F)(F)C(F)(F)C(F)(F)C(F)(F)C(F)(F)C(F)(F)C(F)(F)OC(C(F)(F)F)c1nc(nc(C(F)(F)F)n1)>}
78	Poly(vinyl butyrate)	*CC(OC( = O)CCC)*	{$CC(OC( = O)CCC$}	{$CC(OC( = O)CCC)$}
113	Poly(isobutyl methacrylate)	*CC(*)(C)C( = O)OCC(C)C	{$CC(C)(C( = O)CC(C)C)$}	{$CC(C( = O)OCC(C)C)(C)$}
192	Poly(cyclohexyl α-chloroacrylate)	*CC(*)(Cl)C( = O)OC1CCCCC1	{$CC(C( = O)C1CCCCC1)(Cl)$}	{$CC(C( = O)OC1CCCCC1)(Cl)$}
236	Poly(N-vinyl carbazole)	*CC(*)n1c2ccccc2c2ccccc21	{$CC(N1c2c(cccc2)c3c1(cccc3)$}	{$CC(N1c2c(cccc2)c3ccccc13)$}
283	Poly(quinoxaline-2,7-diylquinoxaline-7,2-diyl-p-terphenyl-4,4′-ylene)	*c1ccc(-c2ccc(-c3ccc(-c4cnc5ccc(-c6ccc7ncc(*)nc7c6)cc5n4)cc3)cc2)cc1	{<c1nc2cc(ccc2nc1)c3cc4nc(cnc4cc3)<, >c1ccc(cc1)c2ccc(cc2)c3c(cccc3)>}	{<c1ccc(cc1)c2ccc(cc2)c3ccc(cc3)c4cnc5ccc(cc5n4)c6ccc7ncc(nc7c6)>}

Subsequently, we compared the BigSMILES generated by our conversion algorithm from SMILES with the corrected ground-truth records. The SMILES data were input into the Python workflow code to generate BigSMILES outputs. Of the generated results, 128 cases matched perfectly with the ground-truth; for the remaining 176 cases, a manual verification process was conducted to confirm whether they represented the same polymer with only differences in ordering. After a meticulous review by a data scientist and chemist, it was confirmed that the conversion process proceeded without errors. The primary focus of this conversion success rate verification was to ensure the accuracy of the transitions between the AA and AB types as well as the representation of ring opening polymerization from asymmetric epoxides. Consequently, it was confirmed that these processes proceeded without errors.

The second procedure involved confirming whether the representation, that had already been converted to BigSMILES using our algorithm, matched the original SMILES when converted back to SMILES. This process was conducted for the entire dataset comprising 4,927,181 records. Through the verification using the programming language Python and the chemical representation package RDKit, it was confirmed that they all accurately represented the repeating unit as in the original SMILES, despite instances wherein the ordering differed in terms of the SMILES syntax. This result indicates that it is possible to use SMILES data converted from BigSMILES as is or after canonicalization.

Finally, we examined whether the conversion to BigSMILES was successful for SMILES representations of the same repeating unit but with varying ordering. This process was undertaken owing to the characteristics of the SMILES representation, as depicted in Fig. 2, which is non-unique yet unambiguous for a perticular structure. Because a single structure may be expressed in various orders, SMILES necessitates such verification. For the 304 existing structures, we generated five different SMILES representations with varying orders using RDKit and input them into the conversion algorithm to check whether they all resulted in the same BigSMILES representation. The results confirmed successful and consistent conversion in all cases, indicating that the conversion algorithm will still work if a SMILES representation is fed into the algorithm without going through the process of refinement using a separate tool.

Through this technical validation process, we demonstrated that our workflow fully supports interconversion between SMILES and BigSMILES representations for homopolymers with two polymerization points, and that the BigSMILES representation of the data we provide is correctly converted and can be used as such.

## Usage Notes

All source code provided was implemented in Python. The experiments were conducted in Python version 3.7.11, and the required packages were specified in the “requirements.txt” file. Within the provided codes, “s2bigs.py” and “bigs2s.py” encompass essential functions. The other codes were the test scripts used to generate the dataset using these functions. Each step of the code was explained through comments. To make these codes user-friendly and usable for practical applications, the source codes were packaged into a Python package named “BigSMILES_homopolymer”. In this section, a brief explanation of the core functions will be outlined. Therefore, for actual application or modification of the code and package, detailed information can be obtained from the provided GitHub repository in which the code is available.

The three functions starting with the name “Data_Load” in the code can import data files in the txt, csv, and xlsx formats. The input files must contain a column containing SMILES information. When importing data in the txt format, the code allows the efficient handling of large datasets by limiting the number of rows and the SMILES length to be imported.

Upon loading the data using the aforementioned data loading functions, it can then be converted globally using the “Converting” functions, divided into 100,000 records and then saved as a csv file in a specified folder location. The “move_parallel” variable in the “Converting” function in “s2bigs.py” was used during the search for two reactive end groups within the same SMILES representation. Setting this number to −1 enabled searching across all SMILES orderings. However, in cases where the data were generated through simulations and not through actual polymers, finding two reactive end groups for all SMILES orderings may be challenging. In such instances, setting this value to zero can enhance processing speed.

Unlike the aforementioned codes that provide mutual conversion between SMILES and BigSMILES, the code for altering versions within BigSMILES representations is declared within the comprehensive file of functions titled “BigSMILES_homopolymer.py.” This code is declared as a class named “version_converter” and currently provides conversion between the officially documented BigSMILES representations, versions 1.0 and 1.1. The versions developed to date, and future BigSMILES syntax additions, if any, are documented on the following GitHub page: (https://olsenlabmit.github.io/BigSMILES/docs/line_notation.html).

## Data Availability

The collected SMILES datasets can be accessed in the corresponding supplementary information files^7,30^ and Zenodo^38^. The Python codes of the SMILES to BigSMILES conversion algorithm, BigSMILES to SMILES conversion algorithm, and examples of the analysis conducted in this study, including technical validations, are available at GitHub: (https://github.com/CDAL-SChoi/BigSMILES_homopolymer).
